# Analysis of Crisis Management of Medical Disputes in China and Australia: A Narrative Review Article

**Published:** 2019-12

**Authors:** Tianlu YIN, Zhaojie LIU, Yanli XU

**Affiliations:** 1. Department of Social Medicine and Health Management, La Trobe University, Melbourne, Australia; 2. Department of Health Management, La Trobe University, Melbourne, Australia; 3. Department of Epidemiology, Hebei University of Engineering, Handan, P.R. China

**Keywords:** Medical dispute, Crisis management, China, Australia

## Abstract

At present, the increasing trend of medical disputes has become a serious problem in the work of medical institutions, and directly affects the social stability and orderly development of the institutions. We searched the literature on medical disputes and crisis management in China and Australia within PubMed (2010–2019), China Knowledge Network (CNKI, 2010–2019), and Wanfang Data Knowledge Service Platform (2010–2019). There are several drawbacks in the management of hospital medical disputes in China: 1) the knowledge of crisis is unilateral and not systematic; 2) there are too much stereotype thoughts in crisis management; 3) the crisis attribution is too simple; the crisis impact assessment is insufficient. It is worth learning from Australia’s system, including the legal system, relevant non-governmental organizations, and doctor-patient communication. In view of the malpractice existing in China’s medical dispute management, Australia’s legal system for handling disputes, doctor-patient communication and other aspects are worthy of our reference. In particular, the construction of third-party supervision and mediation institutions and the prevention of doctor-patient disputes should be optimized.

## Introduction

In recent decades, the number of global medical disputes has increased dramatically (1–2). In response to the high incidence of medical disputes, in 2016, the National Family Planning Committee of China and other nine ministries jointly issued “Notification on the special action plan to severely crack down on crimes involving medical of-fences (National Health Service [2016] No.34). The world famous medical journal Lancet evaluated Chinese medical institutions and medical workers who has been facing the risk of medical disputes and bad medical practice environment by a number of articles like “A new generation of Chinese doctors who are facing a crisis (3)”, “Do Chinese doctors have a future? (4)”. The journal thinks that Chinese medical institutions has become a “dangerous place”, “battlefield”, or “fortress”, and the doctor would become a “high-risk profession”. The worsening relationship between doctors and patient has caused bad effect to contemporary and future medical service provider. In the face of the increasingly high frequency and amount of claims for medical injury, the risk of liability for medical damage has seriously troubled all participants in the diagnosis and treatment activities, and even become a “bottleneck” affecting and restricting the development of Chinese medical and health undertakings (5).

From 1991 to 2005, 7.4% of doctors filed malpractice claims annually, and 1.6 percent received compensation for malpractice. Compared with that in 1991, the number of medical disputes has tripled in 2005 (6). The increasing trend of medical disputes year by year has become a problem troubling the normal operation of medical institutions, and directly affects the social stability and orderly development. Health administrative departments and medical institutions have done a lot of work in mediating, but have not achieved good results (7). In Australia, medical disputes are also common events, but such disputes are often handled smoothly. Besides, there were few malignant medical disputes (8).

This paper summarizes and analyzes the experience of doctor-patient crisis management in China and Australia, and puts forward the adjustment direction of medical dispute crisis management in China in the future.

## Results

### Crisis management of doctor-patient disputes in China

#### Current situation of doctor-patient disputes

Since the beginning of the 21st century, the number of medical disputes in China has increased rapidly due to the contradiction between the supply and demand of medical services, as well as the enhancement of patients’ awareness of safeguarding their rights and the imperfection of the legal system. There were even “medical dispute profiteers” (9, 10). The incidence of medical troubles has been also increasing year by year, including economic losses and personal injuries of medical workers. In 2010, 17,243 medical disputes occurred in China, increased by nearly 7,000 compared with five years ago (11).

The incidence of doctor-patient disputes is high according to the investigation report “The situation of violence in hospital places” by Chinese hospital association. The number of hospitals where doctors were injured jumped from 47.7% in 2008 to 63.7% in 2012. In 2008, there were 20.6 incidents of abuse and threats against medical staff in each hospital, and the number reached 27.3 in 2012 (12). The conflict between doctors and patients is becoming more and more intense. According to the data released by the Supreme People’s Court of China in May 2015, in 2014, courts nationwide concluded 155 cases of violent killing and wounding of doctors. “White paper on the practice of Chinese doctors” mentioned: according to the research results in 2014, 59.79% of medical staff have been subjected to verbal violence, 13.07% have been physically injured, and only 27.14% have not experienced violence (13). According to the date from Chinese Ministry of Health, there were 73 million outpatient visits and about 70,000 medical disputes in all medical institutions nationwide in 2015. Although the proportion is not high (9%oo), each dispute increases the gap between doctors and patients (14).

In 2002, China promulgated the relevant laws to control the medical disputes (15). Although the problem between doctor and patient can be negotiated, we have to admit that some gap between doctors and patients in medical disputes impeded solving the problem. On the contrary, it could deepen the contradiction between the two sides (16). According to Article 46 of *Medical malpractice processing law* of China, when civil liability disputes of compensation for medical malpractice occur, there are three ways to solve them: First, consultation between doctors and patients; Second, the parties apply for mediations in the administrative department of public health; Third, directly bring a civil lawsuit to the court (17). On April 1, 2019, the Supreme People’s Court of China put *The provisions about the civil action evidence* on force.

#### The reason

First, policies and regulations are not sound, medical disputes lack of legal support in the disposal. For example, The regulations on the treatment of medical accidents stipulates that “medical institutions shall not be liable for compensation if they do not belong to medical accidents”, while the *General principles of the civil law* stipulates in paragraph 2 of article 106 that citizens and legal persons shall bear civil liabilities if they infringe upon other people’s property or person through their faults. In appraisal orgnaization choices, *Notice about trial of medical disputes in civil cases according to the <regulations dealing with medical malpractice>by SPC* stipulates: medical treatment malpractice undergoes appraisal by specialists from medical association, the other medical treatment compensation dispute identifies by judicial appraisal organization except medical treatment malpractice. There are effect problems, for example, the hospital provides evidence like formatted informed consent, many patients or family members do not fully understand the content, or have no time to read the content in emergency, or have no choice and had to sign. This kind of special professional for medical industry and format customization on medical documents caused the unequal information between doctors and patients as well as litigation difficulty.

Second, the medical expenses are too heavy, which increases the difficulty of handling medical disputes. According to statistics, due to the uneven distribution of medical resources and the limitations of medical technology in primary hospitals, tertiary and secondary hospitals account for 74% of medical resources, causing overcrowding in large hospitals, and medical disputes (97.5%) occur in tertiary and secondary hospitals (18). Although Chinese market transformation since 1978 has promoted the rapid development of medical and health services, it has also led to an increase of medical costs, the serious shortage of public health and medical resources has still not been improved. There are still 300 million people having no health insurance (19). Another serious problem is the uneven distribution of medical resources in different regions. Hospitals in big cities have well-trained clinicians and advanced medical equipment, while hospitals in small towns and rural areas lack medical personnel and basic medical equipment.

Third, the staff have limited ability to deal with medical disputes and cannot properly solve medical disputes in a timely manner, and the mass media exaggerated the doctor-patient disputes. After the occurrence of doctor-patient disputes, managers have no basic legal protection, and patients have no better channels to safeguard their own rights and interests. As a result, the problems between health workers and patients became more serious, and the crisis was not contained in the bud, which led to a larger crisis (20). Fourth, the lack of specialized management departments. When solving the crisis of doctor-patient dispute, there is no institution with a strong system for supervision and management. When dealing with such problems, most departments will choose to escape and shirk responsibility, and eventually the conflicts will be ignored. Fifth, excessive media coverage. When the media report disputes in hospitals, most of them exaggerate the problems of medical workers, including “poor professionalism”, “bad attitude” and other problems (21). As a result, the image of medical institutions in people’s mind is negative Sixth, doctor mishandling. Among 76 cases of orthopedic medical disputes, iatrogenic was the main cause of medical disputes, accounting for 65.79% (22). On the one hand, because the department of orthopedics mainly deals with emergency surgery, the doctors do not have enough time to explain the patient’s detailed condition clearly to the patient and family members; On the other hand, some medical staff lack enough patience and responsibility to explain the possible accidents and matters needing attention before, during and after the operation to patients and their families in detail. The main non-iatrogenic causes were the patients and their families’ failure to follow the doctor’s advice and postoperative complications (22).

#### Research on crisis management of medical disputes in China

The research on crisis management in China lags behind compared with that in western countries. The research on medical dispute crisis management is in a beginning stage. After the outbreak of SARS in 2003, Chinese government and scholars began to pay attention to crisis management in the field of public health. According to the research results in the past decade, crisis management has been involved in various fields in China In 2011. Crisis management of medical disputes can help hospitals change medical disputes from “emergencies” to “pre-occurrence” events, grasp information in advance, and change their thinking from “post-emergency treatment” to “pre-occurrence control” 23). Based on the crisis management theory, it is necessary to establish a unified early warning standard and system for medical disputes, so as to prevent and reduce the occurrence of medical disputes in medical institutions (24). The hospital should set up the corresponding complaint reception personnel to report the possible medical disputes to the leadership in the form of “alert”, so as to prevent the occurrence of medical disputes with acute crisis awareness (25). Multi-level crisis public relations system can effectively prevent and deal with medical disputes of patients with sudden death after surgery, which is worthy of promotion and application (26). The multi-level crisis public relations system in this study is composed of the principles of pre-training, intervention and communication in the event, and post-rectification and assessment. The implementation is completed by the public relations systems of the relevant departments, doctor-patient offices, security departments, lawyers and hospital leaders (26).

### The malpractice existing in crisis management of hospital medical dissension in our country

#### Too one-sided and unsystematic understanding of the crisis

The crisis before the outbreak, the risk assessment and crisis prevention work have been ignored, which make the hospital management department unable to predict the crisis timely, and unable to improve the quality of the management of the hospital medical disputes. It is not conducive to the hospital medical disputes on the solving the problem (27).

#### The characteristics of crisis handling mechanization are too obvious

Medical disputes in Chinese hospitals are mainly handled by the medical department, coordinated by the security department. They failed to found a joint management team with other relevant departments. To a certain extent, after the occurrence of medical and health disputes, the medical service department did not deal with medical disputes in a scientific manner according to the characteristics and specific circumstances of the crisis in time, which has an adverse effect on the handling the crisis. At the same time, the application of crisis management mode can easily lead to a solid thinking pattern of crisis management staff, which makes the hospital being the passive party in doctor-patient disputes and unable to truly solve the doctor-patient relationship.

#### Insufficient impact assessment of crisis

In dealing with medical disputes, it usually takes more time and energy to investigate the facts and analyze the causes, while ignoring the catalytic role of time cost and media in crisis events. As a hospital manager, it is often implied that disputes are caused by patients’ excessive expectations, while patients think that the high cost has not achieved the desired effect. Both sides always see the problem from different angles and get involved in long-term mediation or litigation, the cost is higher to protect their legitimate rights and interests than the cost of solving the problem. Crises are apt to lead to malignant events. The best solution to a crisis is “Symptomatic treatment” (27).

### Medical dispute crisis management in Australia

#### System

In Australian, one of the departments is responsible for quality management, its purpose is to check the service quality of medical institutions. It also has a department responsible for drug supervision, it ensures that the drug use is implemented in accordance with national standards (28). It aims at regulating medical workers to improve the quality of health services and ensure normal operation and reduce the medical risks of patients in the treatment process.

#### Non-governmental organization

There are many non-governmental organizations or associations in Australia, many of which are public welfare organizations. The government may have many disadvantages in the implementation of medical risk management, and some organizations start to appear to help solve medical problems. Australia is very supportive to the work of such non-governmental organizations. The organization can work with health care providers to improve the quality of care. Australia has a special committee to certify these organizations. The NGOs after certification are generally professional, which could improve the service quality and reputation of medical institutions (29).

#### The law

In Australia, legal efforts were made to improve the medical level and service quality of the whole institution to a greater extent, so as to reduce the possibility of medical dispute crisis (8, 30).

In Australia, the doctor cannot give a death notice within 24 hours for deaths due to a sudden accident, the death of a mental patient or death due to anesthetic drugs, etc. They must report this situation to the police station. After investigation, the health consultant identifies the death caused by special circumstances or a death caused by medical malpractice. Once confirmed, the court will make corresponding punishment or approval. The liability determination of medical disputes and accidents in Australia is also partly completed by the ethics committee within the hospital, and the other part is determined and arbitrated by the judicial system. The court has a special “health consultant”. Australian hospitals have at least one or two ethics committees. An ethics committee will surely be set up to determine and deal with the responsibilities of various medical malpractices and solve the contradictions therein. Australia’s relatively complete medical insurance and government compensation mechanism also allow doctors and patients to be relatively calm in handling accidents and disputes, and willing to trust the conclusions reached by relevant institutions.

In Australia, every doctor will buy professional insurance, when the amount of compensation awarded by the court due to medical disputes exceeds 1 million Australian dollars, the government shall bear the excess. Because of occupational insurance and the government’s system of excessive compensation, medical disputes in Australia tend to be mild.

## Discussion

### Third party supervision and mediation institutions should be added

The Australian experience can be referred. The third-party agencies, third party mediations are more neutral than administrative mediation, and more authoritative than bilateral negotiations. There are many reasons that may lead to medical disputes, such as the problems of doctors or patients themselves, communication problems and so on. Resolving medical disputes through litigation not only takes up a lot of judicial resources, but also has high cost, complicated procedure and strong antagonism. It is one of the effective ways to alleviate the contradiction between doctors and patients and solve medical disputes through mediation. With the enhancement of people’s awareness of safeguarding their rights, they are more and more eager to have a relatively neutral third-party organization to coordinate and handle conflicts and disputes, so as to ensure a more fair and just result of mediation. The Commission was born in this background.

### Optimize the evaluation system for maintaining social stability and give overall consideration to the relationship between maintaining stability and safeguarding rights in accordance with the law

Although the individual interests of some patients and their families are legitimate, their behavior norms, methods and procedures should also be legal. As for the conflicts caused by medical disputes, the government departments should control the behaviors of both parties in the conflict within the scope permitted by law, support patients and their families to protect their legal rights, and stop illegal activities in time. There is no conflict in law between safeguarding social stability and punishing illegal rights and interests, When dealing with problems, only by strictly implementing laws and regulations can the law enforcement departments of the government fairly solve the problems, enable citizens and social organizations to revere the law and form a basic law-abiding mindset of abiding by the law in the whole society. If only the interests and risks of the departments are taken into consideration and laws, and regulations are ignored, the public will lose balance and the society will lose order, thus causing greater instability.

### Do a good job in sound laws and regulations on medical disputes

It is necessary to improve the system of laws and regulations governing the handling of medical disputes between doctors and patients, and adjust existing laws and regulations that conflict with or are incompatible with the civil law and the law on tort liability. Given the implementation of compulsory medical liability insurance could achieve the purpose “to protect the damage of medical treatment accident victims get compensation according to law, to promote medical security”, based on the principles of legal reserve requirements, it is suggested that China should formulate a unified administrative “compulsory medical liability insurance regulations” (5).

### Further strengthen the popularization of medical science knowledge, optimize the format of informed consent, and strengthen the effect of doctor-patient communication

Let the public know that in the face of disease diagnosis and treatment and rescue, Medical institutions has accumulated much experience in human medical cognitive practice and diagnosis and treatment ability. The relevant laws of disease diagnosis and treatment and the relative certainty between different medical activities were also observed.

### Optimize the medical service system, let doctors and patients from “one-stop relationship” to “contract relationship”

In order to solve the disputes between doctors and patients, China has introduced various policies and improved various mechanisms in recent years. The relevant personage of the state health commission showed that China is speeding up the formation of a coordination mechanism formed by public security, protection and supervision, comprehensive governance, publicity and other departments. In 2015, the medical disturbance behavior was included in the criminal law regulation. Meanwhile, the medical liability insurance system has been established and improved, by the end of 2016, 110,000 medical institutions had participated in medical liability insurance. This is not only conducive to dispersing the economic and occupational risks of hospitals and medical staff, but also conducive to forming reasonable expectations of both parties.

In addition, “hierarchical diagnosis and treatment” enables a “long-term contract” relationship between family doctors and patients, rather than the current “one-stop” relationship, which is also conducive to reducing the occurrence of disputes. Patients are guided by the family doctors “where to find a doctor and which doctors they should see”, instead of “blindly flocking to big hospitals”.

### Simplify relief procedures

In the judicial remedy, some simple disputes can be solved by judicial summary procedure. In the process of litigation, the damages caused by medical risks such as complications and medical accidents should be fully considered within the scope of compensation, to guide patients and their families through legal rights to obtain their compensation. The administrative relief realizes its function through the doctor-patient dispute mediation committee. The committee may be established through the bureau of justice, an insurance company or a political and legal commission. The members may be judges, medical personnel, supervisory commissions, government functionaries or members of the public. In mediation, the credibility can be enhanced by strengthening the mediation function, simplifying the mediation process, improving the procedure supervision, and highlighting the mediation effectiveness and execution. Social relief brings material help or spiritual relief to patients or their families through public power. It plays an important and irreplaceable role in correcting “market failure”, adjusting resource allocation, realizing social fairness, maintaining social stability and building a harmonious socialist society (31). It can be realized by social organizations, people, civil affairs departments and media, However, in the application, the government needs to strengthen publicity and supervision, guidance and control, to prevent the emergence of some confusing public opinion.

## Conclusion

In view of the malpractice existing in China’s medical dispute management, Australia’s legal system for handling disputes, doctor-patient communication and other aspects are worthy of our reference. In particular, the construction of third-party supervision and mediation institutions and the prevention of doctor-patient disputes should be optimized.

## Ethical considerations

Ethical issues (Including plagiarism, informed consent, misconduct, data fabrication and/or falsification, double publication and/or submission, redundancy, etc.) have been completely observed by the authors.
